# The Causes of Disease

**Published:** 1917-11-17

**Authors:** 


### The Hospital

The Workers' Newspaper of Administrative Medicine and Institutional Life, Administration, National Insurance and Health.

No. 1641, Vol. LXIII. SATURDAY, NOVEMBER 17, 1917. PRICE ONE PENNY

### THE CAUSES OF DISEASE.

The Bradshaw lecture on "The Causes of Disease," recently delivered before the Royal College of Physicians by Dr. Ernest Reynolds, revives a note which was sharply emphasised in former days but which in recent times has been sounded much less strongly. Text-books dealing with systematic medicine used invariably to open their pages with such a discussion, and causes, "predisposing" or "exciting," were duly ranged and confidently presented as responsible for various definite and specific effects. The enterprise necessarily involved the question, What is a cause? And into this issue the more philosophical authors and lecturers did not shrink from entering. Far be it from us to suggest that in all this there was not an influence of some educational value in the mental training of the student. Yet the practical value of it all as an aid in dealing with an individual patient certainly did not lie on the surface. General propositions regarding the influences prejudicial to health arising from such agencies as age, sex, cold, heat, climate, occupation, food, alcohol, and the rest, might be quite true, but experience showed so many exceptions to each individual rule that the doctrine remained largely in the air and retained only a weakening hold on the practitioner's mind. To satisfy the not unnatural curiosity of a patient, or to attract the confidence which attaches itself to an unhesitating display of knowledge, the general proposition could be applied—and in this summary fashion—to the individual instance. But this does not mean that it carried conviction to the mind of the practitioner or that it threw light on his responsibilities.

The discussion, on this method, of the causes of disease had yet another defect. It was not sufficiently exhaustive. Too vague to be of much practical service it was, on the other hand, not pushed to a point where it could afford a mental resting-place. Clearly, to reach such a position, two conditions must be satisfied. The inquiry must show us not only that certain agencies cause particular diseases, but must also reveal in detail the chain of causation, for, otherwise, we are left merely with hard facts about the relations of which we have nothing but an arbitrary statement. Still further, if diseases are evils, as will generally be admitted, and if we must embark upon a study of their causes, we cannot in strict reason halt halfway. A study of causes in the comprehensive sense of the term means a study of origins, and a study of the origins of particular evils cannot well avoid the question of the origin of evil in general. Whether any ambitious medical author or lecturer has been sufficiently thorough to push his study of the causes of disease back to this remote point we do not know. The majority certainly have been content with a more limited conventional and formal excursion, and if there has been an exception he has been mute and inglorious, for the mystery of the origin of evil still remains a standing perplexity to the human mind. Altogether, it cannot be said that the classical inquiry into the causes of disease has afforded either much mental illumination or much practical service.

With the arrival of bacteriology a more confident and precise opportunity appeared to present itself. That certain diseases were associated with the presence of micro-organisms in the blood or tissues was a matter of demonstration. Here, surely, at long last, we held the "causes" of these diseases. Further, obvious analogies suggested that other diseases must have a kindred causation. Hence a vigorous hunt for germs was aroused, and in not a few instances the quarry was run to earth. Yet, without much delay, the voice of the sceptic began to assert itself. According to its more extreme accents the germs were maliciously affirmed to be not "causes," but mere "concomitants." A less destructive criticism suggested that while indeed the germs might be the "seed," their efficacy was *nil* unless there was provided for them a suitable "soil," and obviously the more widely spread the germs were shown to be the less became the virtue of their claim to be the "sole cause" of the diseases they respectively represented, for not all persons exposed to their influence became their victims. Thus the fair promise of simplification of the story of disease causation offered by the advent of bacteriology faded away, or, if it provided a ray of light it did not bring the expected blaze of the sun at noonday. The germs were something in the matter, it was true, but the inquiry was still left to face the great mystery—the man himself. Not everyone who suffered the germs developed the disease, and though many were present in Siloam the falling tower slew but a limited number. Thus the causation, even of diseases associated with the presence of micro-organisms, was found to be a highly complex study which has led the inquirers into such involved problems as personal idiosyncrasy and immunity, and has been fruitful in ingenious hypotheses and bold speculations. Moreover, not even a pathologist will affirm that all diseases are due to micro-organisms, and the excluded group obviously are untouched by the considerations just mentioned.

The record as here stated has, it must be admitted, a very pessimistic quality. For the great aim in reference to diseases is to prevent them, and successful prevention can hardly be anticipated in the absence of a knowledge of etiology. The practical situation, however, is a less gloomy one. Something has certainly been learned of the conditions which severally favour or hinder the development of some diseases, as, to quote but a single instance, the limitation of epidemics in our armies clearly shows; and the value of this knowledge to humanity has been great. But, taking the field as a whole, it is largely wrapped in obscurity. In the absence of secure knowledge there is a distinct danger that many schemes of prevention now confidently advanced will come to nought, and the direction in which enterprise and investigation should first be directed is thus plainly indicated.

